# A high content, high throughput cellular thermal stability assay for measuring drug-target engagement in living cells

**DOI:** 10.1371/journal.pone.0195050

**Published:** 2018-04-04

**Authors:** Andrew J. Massey

**Affiliations:** Vernalis Research, Granta Park, Cambridge, United Kingdom; Brandeis University, UNITED STATES

## Abstract

Determining and understanding drug target engagement is critical for drug discovery. This can be challenging within living cells as selective readouts are often unavailable. Here we describe a novel method for measuring target engagement in living cells based on the principle of altered protein thermal stabilization / destabilization in response to ligand binding. This assay (HCIF-CETSA) utilizes high content, high throughput single cell immunofluorescent detection to determine target protein levels following heating of adherent cells in a 96 well plate format. We have used target engagement of Chk1 by potent small molecule inhibitors to validate the assay. Target engagement measured by this method was subsequently compared to target engagement measured by two alternative methods (autophosphorylation and CETSA). The HCIF-CETSA method appeared robust and a good correlation in target engagement measured by this method and CETSA for the selective Chk1 inhibitor V158411 was observed. However, these EC_50_ values were 23- and 12-fold greater than the autophosphorylation IC_50_. The described method is therefore a valuable advance in the CETSA method allowing the high throughput determination of target engagement in adherent cells.

## Introduction

A critical component of small molecule drug discovery is determining and understanding ligand-protein interactions (target engagement) at the site of drug action in the cell. For a large number of potential drug targets, “classical” approaches (e.g. monitoring changes to substrate or product generation) are not amenable. The cellular thermal shift assay (CETSA) first described by Martinez Molina *et al* [1] has become frequently used in target engagement studies. The assay relies on the principle that ligand binding results in thermal stabilization (or sometimes destabilization) of the bound protein. Practically, the CETSA method measures the amount of soluble protein remaining in cells following heating at various temperatures in the absence or presence of a ligand. The classic method [1,2] relies on treating cells with ligand and then heating in suspension at relatively high densities (of the order of 1-3x10^7^/mL) in a thermocycler. Following cell lysis, cell debris as well as aggregated and precipitated proteins are removed and the remaining soluble protein detected by, for example, western blotting or homogenous detection methods (e.g. AlphaScreen, ELISA, referred to as HT-CETSA etc.) [2,3]. As this method does not rely on modification of either the target or an interacting ligand, it can in theory, be applied to any target in any cellular system. A recent advance has seen the application of high-resolution mass spectrometry to the whole proteome enabling not only the measurement of desired on-target effects but also the identification of potential off-target liabilities [4–7].

For adherent cells, the requirement to heat the cells in suspension at high density is an obvious drawback and the process of trypsinization and resuspension may alter cellular physiology and target pharmacology. Additionally, having to treat cells at high cell densities may result in an underestimation of target engagement potency and make comparisons to downstream pharmacology assays more difficult. As the CETSA method determines the amount of soluble, folded protein remaining, we hypothesized that heating cells growing attached to a 96 well plate (96WP) and determining changes in the amount of target protein still correctly folded by high content immunofluorescent imaging might be a useful adaption of the CETSA method for adherent cells. We have therefore developed a novel cellular target engagement assay in adherent live cells using the principle of ligand-induced changes to protein thermal stability coupled with high-content single cell immunofluorescent imaging in an attempt to mitigate some of these potential liabilities.

## Materials and methods

### Cell lines and cell culture

HT29 and U2OS cells were purchased from the ATCC and grown in DMEM or McCoys 5a media supplemented with 10% fetal bovine serum (Thermo Fisher Scientific) and 1% penicillin / streptomycin at 37°C and 5% CO_2_ in a humidified incubator. Cell lines were established as a low passage cell bank and then routinely passaged in our laboratory for less than 3 months after resuscitation. Authentication was by STR profiling (LGC Standards).

### Plate heating and temperature determination

Temperature changes in 96 well plates or PCR tubes was determined with two K type hermetically sealed thermocouples (TWHSEK00001M0AP7, Sterling Sensors, UK) connected to a 2TC dual-channel thermocouple thermometer WIFI measuring module (Papago). Temperatures were logged using SNMP Data Logger software (AGG Software). The K type thermocouples and water bath temperatures were calibrated with a pre-calibrated Fisher digital thermometer (11719715, Fisher Scientific Traceable).

Plates were heated as follows: (i) sealed with adhesive aluminum PCR plate foil and then floated or submerged in a water bath (Grant Instruments W14) containing water pre-heated to the desired temperature or (ii) 1 L of DPBS containing magnesium and calcium (D1283, Sigma) was placed in a clean water bath (SUB6, Grant Instruments) and heated to the required temperature. The media was removed from the wells of the CellCarrier plate and the plate submersed in the DPBS so that all wells were full of DPBS and the plate was not touching the bottom of the water bath. The plate was incubated in the DPBS for 3 minutes, removed, the hot DPBS removed and replaced with 100 μL room temperature DPBS and then incubated at room temperature for 5 minutes. In some experiments, the DPBS was substituted for live cell imaging solution (LCIS, 20 mM Hepes, 140 mM NaCl, 2.5 mM KCl, 1.8 mM CaCl_2_, 1.0 mM MgCl_2_, pH 7.4).

For temperature determination experiments, the thermocouples were placed in two different wells (one edge, one center of plate) and the hole sealed with blu-tack and parafilm (S1 Fig).

### Reagents and antibodies

Chk1 inhibitors were provided by Vernalis Research or purchased from Selleckchem and prepared as a 20 mM stock in DMSO, aliquoted and stored at -20°C. The same DMSO stock was used for all experiments. The antibodies and the dilutions used in these studies are listed in Table 1. The selectivity of the Chk1 antibodies was confirmed using an ON-TARGET plus Human CHEK1 SMART pool siRNA (GE Dharmacon) transfected with Lipofectamine RNAiMAX (Thermo Fisher Scientific).

**Table 1:** List of antibodies used.

Target	ID.	Supplier	Prod. Code	Spp.	Appl.	Dil.
Chk1	2G1D5	CST	2360	M	IF	1:200
					WB	1:5000
Chk1	G-4	Santa Cruz	sc-8408	M	IF	1:200
Chk1	EP691Y	Abcam	Ab40866	R	IF	1:500
Chk1	FL-476	Santa Cruz	sc-7898	R	IF	1:200
Chk1	N19	Santa Cruz	sc-7235	G	IF	1:200
Chk1	Bethyl	Bethyl Labs	A300-162A	G	IF	1:200
pChk1 (S296)		Abcam	Ab79758	R	WB	1:2500
pChk1 (S317)		CST	12302	R	WB	1:2500
					IF	1:800
pChk1 (S345)		CST	2348	R	WB	1:2500
					IF	1:100

ID., identifier; Appl., application; WB, western blot; IF, immunofluorescence; Spp., species; Dil., dilution; CST, Cell Signaling Technology

### Immunoblotting

Immunoblotting was conducted as previously described [8,9].

### Single cell immunofluorescence

Single cell immunofluorescent imaging was conducted as previously described [8,9] in CellCarrier 96WP or CellCarrier Ultra 96WP (6005550 or 6055300, Perkin Elmer) and imaged on an Operetta high content imager (Perkin Elmer) using a 10, 20 or 40x objective then analyzed using Harmony software (Perkin Elmer).

### CETSA by immunofluorescence (HCIF-CETSA)

10 000 cells were plated per well of a CellCarrier Ultra 96WP (6055300, Perkin Elmer) and allowed to adhere overnight. Compounds were serially diluted in DMSO to 200-times the final assay concentration then diluted 1:200 in media (without FCS). The cell media was removed and replaced with 25 μL of compound in media and incubated at 37°C, 5% CO_2_ for 10 minutes. The plate was sealed with an aluminum plate seal and heated by floating in a pre-heated water bath (Grant Instruments W14) for 3 minutes. 75 μL of room temperature media (without FCS or compound) was added and the plate incubated at room temperature for 5 minutes. Cells were fixed by the addition of 100 μL paraformaldehyde (7.4% in PBS) at room temperature for 15 minutes. Cells were washed 3 times with PBS and Chk1 detected with anti-Chk1 rabbit EP691Y monoclonal antibody (Ab40866, Abcam) and an AlexaFluor488 anti-rabbit secondary antibody (A-11034, Thermo Fisher Scientific). Plates were imaged on an Operetta high content imager using a 20x objective and analyzed using Harmony software.

## Results

### Methods for effectively heating cells in an imaging compatible 96 well plate

The standard CETSA method (CETSA classic) utilizes a thermocycler (or alternatively a water bath) and thin-walled PCR tubes to ensure the rapid, consistent heating and subsequent cooling of the sample. 96WPs suitable for adherent cell growth and high content imaging were incompatible with heating in a thermocycler. We therefore evaluated alternative methods of heating adherent cells in imaging compatible plates. To accurately monitor in well temperature changes, two hermetically sealed, wire k-type thermocouples (approximately 2 x 1mm) coupled to a Papago thermometer WIFI module and data logging software was utilized (S1 Fig). The relatively small dimension of the thermocouple allowed temperature changes in 200 μL volumes to be measured accurately and reproducibly every approximately 0.5–1 second when coupled with the Papago WIFI thermometer.

**Fig 1:**
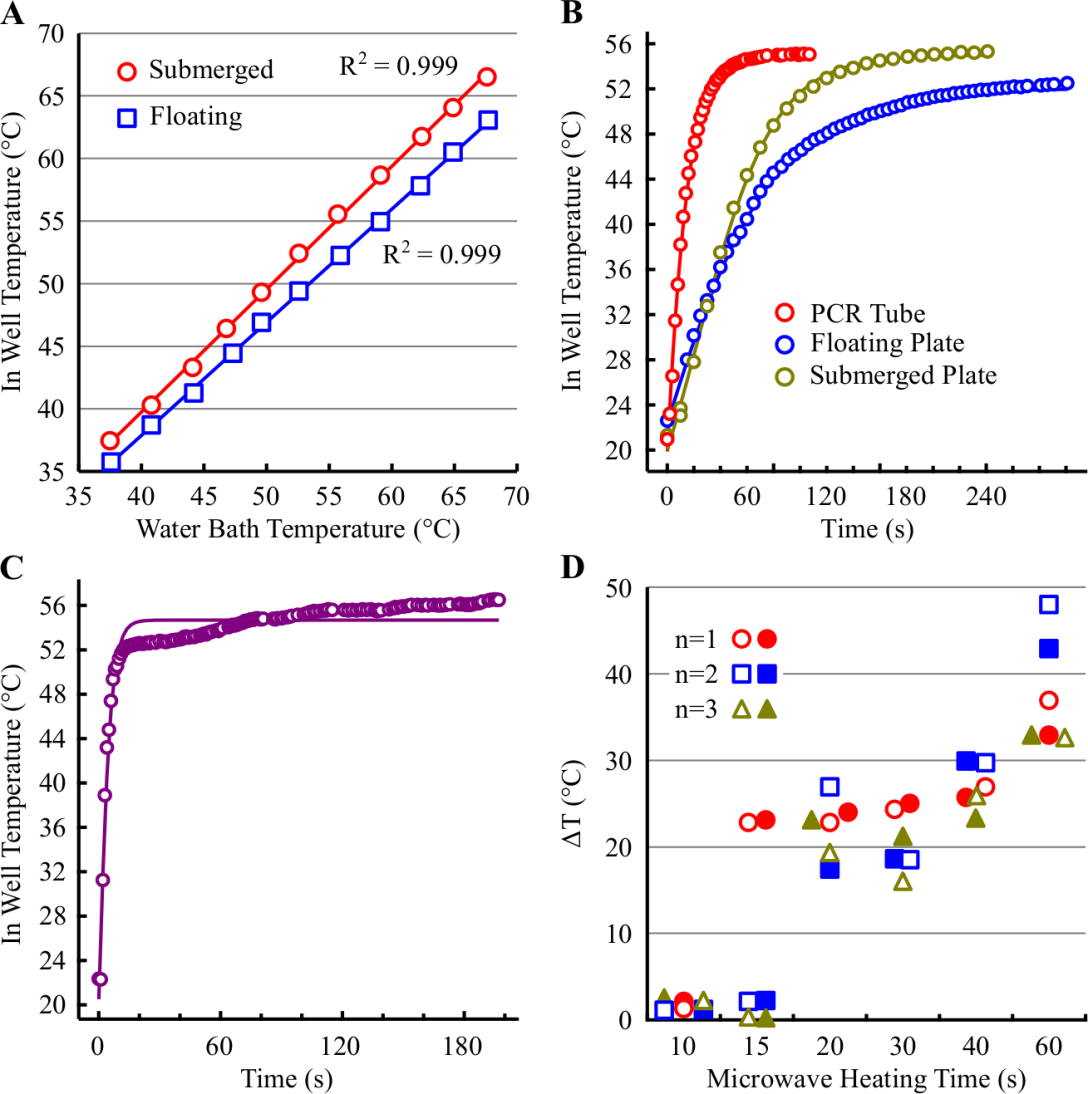
Evaluation of methods for heating 96 well plates. (A) A CellCarrier-96 plate containing 200 μL PBS per well was sealed with an aluminum plate sealer and either floated or submerged in a water bath. The maximum temperature achieved in the well was determined using a K-type thermocouple and are the mean of a central and edge well. The water bath temperature was monitored with a calibrated thermometer. (B) A water bath was pre-heated to 55°C and the temperature monitored over time in the following vessels: a floating 0.5 ml PCR tube containing 200 μL PBS; a floating or submerged CellCarrier-96 plate containing 200 μL PBS per well. Values are the mean of either 2 different PCR tubes or a central and edge well. (C) An unsealed CellCarrier-96 plate was immersed in a water bath containing PBS pre-heated to 55°C and the temperature of the PBS in the well monitored over time with a K-type thermocouple. Values are the mean of a central and edge well. (D) A CellCarrier-96 plate containing 200 μL PBS per well was heated for the indicated times using a standard domestic microwave set to low power. The maximum temperature achieved in a central (open symbol) and edge (filled symbol) well was determined using a K-type thermocouple.

We initially determined the maximal temperature achievable in a sealed CellCarrier 96WP containing 200 μL PBS per well either submerged or floated on top of a pre-heated water bath. The in well temperature achieved in the submerged plate was equivalent to that of the heating water bath and linear over the range of temperatures tested (R^2^ 0.999, Fig 1A). In comparison, the temperature achieved in the floating plate was consistently lower (ΔT 1.9–4.7°C) than that of the heating water bath. Like the submerged plate, the relationship between water bath temperature and in well temperature was linear over the range of temperatures tested (R^2^ 0.999). With either heating method, there was no difference in the in well temperature between a central and an edge well.

The time required to reach the target heating temperature was subsequently determined in a thin-walled PCR tube floating in a pre-heated water bath. The PBS in the PCR tube heated up rapidly reaching a T_max-1_°C in 44–52 s (Fig 1B and Table 2). The time required to reach T_max-1_°C was 3- and 5.5-fold slower in a CellCarrier plate submerged or floating in a pre-heated water bath. Evaluation of the heating properties of different 96WP types (CellCarrier Ultra, Isoplate or round-bottomed) revealed similarly slower time-to-target temperature than observed with a PCR tube (Table 2). Likewise, heating the plate on an aluminum dri-block did not improve the heating performance compared to water bath heating.

**Table 2:** Heating characteristics of 96 well plates.

Plate / Tube Type	Heating Method (Sealed Plate)		Temperature of heating device (°C)		T_max_ (°C)	Time to T_max-1_°C (s)
0.5ml PCR Tube	Water bath	Floating		46.9	46.3	52
				49.7	49.1	44
				52.6	52.1	48
				55.9	55.0	45
Isoplate 96WP	Water bath	Floating		55.7	49.6	703
Round-bottomed 96WP	Water bath	Floating		55.7	50.5	264
CellCarrier 96WP	Water bath	Floating		55.8	52.8	251
CellCarrier Ultra 96WP	Water bath	Floating		55.5	53.0	252
CellCarrier 96WP	Water bath	Submerged		46.8	46.6	152
				49.7	49.6	162
				52.4	52.4	133
				55.7	55.4	140
CellCarrier Ultra 96WP	Water bath	Submerged		55.5	54.9	225
CellCarrier Ultra 96WP	Al dri-block	On top		58.0	53.9	383

96WP, 96 well plate. T_max_, maximum temperature achieved in the plate well / tube. Time to T_max-1_°C, time (in seconds) to reach a temperature 1°C below the T_max_.

To improve the rate of cell content heating, we evaluated the in well temperature-time response profile of an empty, unsealed CellCarrier plate in a water bath containing pre-heated PBS. In this scenario, the in well temperature increased rapidly from 22°C to 52°C in 9.8 ± 1.7 s (n = 4, mean ± SD). The temperature then increased slowly over the next 170 s reaching a temperature approximately 2°C (56.9 ± 0.9°C) higher than the water bath set temperature of 55°C (Fig 1C). Addition of pre-heated PBS at 55.7°C to an empty CellCarrier plate at room temperature resulted in a maximal in well temperature of 45.9 ± 0.3°C (n = 3), a temperature loss of 9.9 ± 0.3°C.

Finally, we evaluated the effectiveness of a standard domestic microwave (of the kind found in may laboratories used to heat agar, agarose etc.) to heat 200 μL PBS in a CellCarrier plate. The maximum temperature achieved in a given well following various heating times with a low power setting was highly variable and non-linear with time (Fig 1D). Additionally, the maximum achieved temperature varied considerably between a central and edge well. Microwave heating was deemed to variable and therefore unsuitable for accurate, reproducible plate heating. We found that immersion of the open plate in pre-heated PBS generated a heating profile that most closely resembled the thermocycler / PCR tube though this too was not without issues. Ideally, a thin-walled 96WP that fits in a thermocycler with a thin, flat bottom compatible with imaging applications is required.

### V158411 does not increase Chk1 thermostabilization in intact cells following immersion in pre-heated PBS

**Fig 2:**
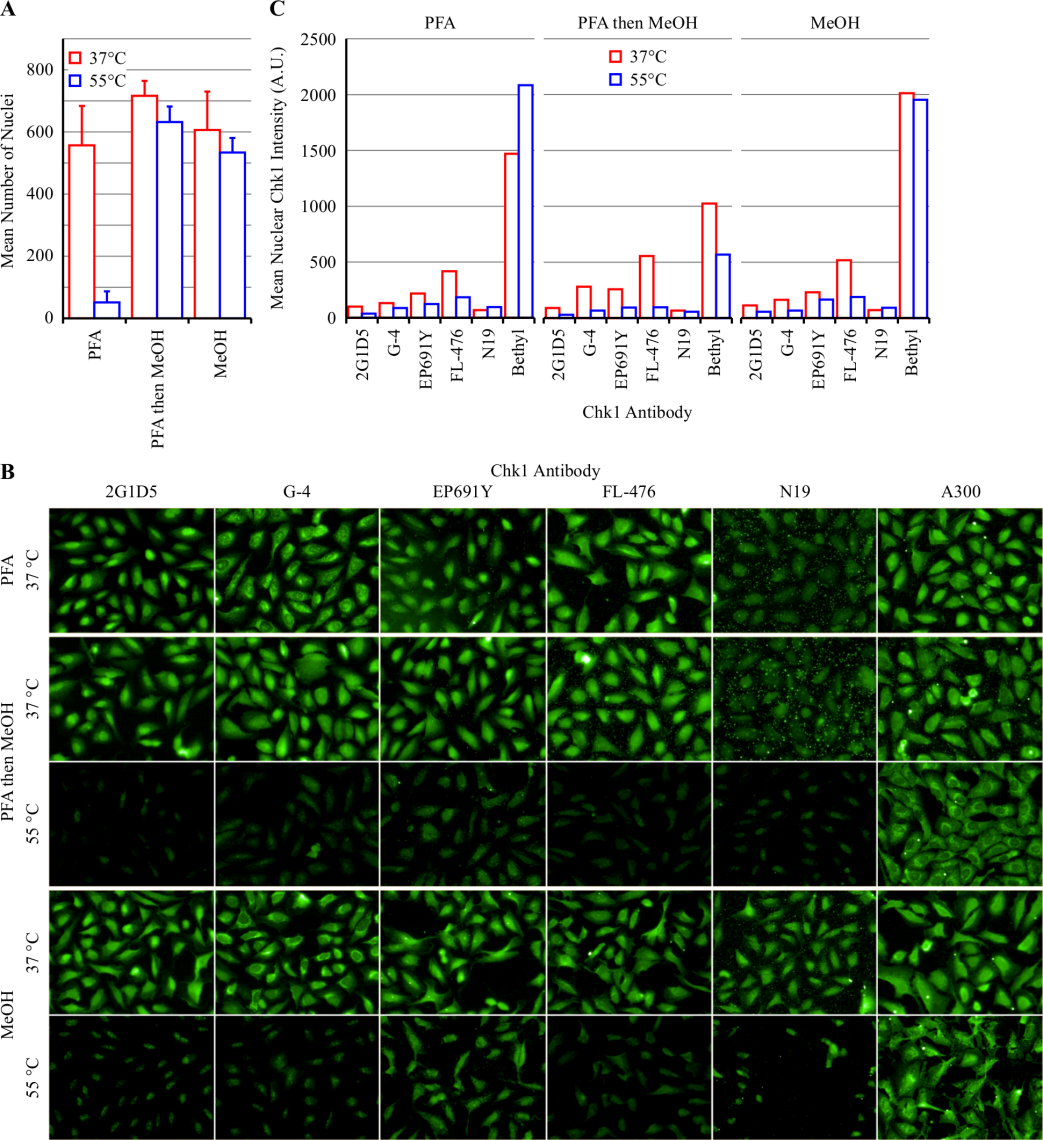
Comparison of fixation methods of heated U2OS cells. (A) U2OS cells were heated using the PBS immersion method in pre-heated PBS for 3 minutes at the indicated temperature, then cooled to room temperature for 5 minutes and then fixed with either paraformaldehyde (PFA), PFA then MeOH or MeOH. Nuclei were stained with Hoechst 33342 and quantified by immunofluorescent imaging. Values are the mean of 12 wells ± SD. (B) Chk1 localization was determined by immunofluorescent staining and high content imaging. (C) The mean nuclear Chk1 intensity was quantified from the images obtained in B. Values are the mean of 2 wells.

Previous work has demonstrated that the selective Chk1 inhibitor V158411 [10] stabilizes Chk1 in HT29 and U2OS cells following heating using the CETSA classic method and western blot detection [11]. As CETSA detects the amount of folded, soluble protein remaining following cell heating, we hypothesized that immunofluorescence and high content imaging may be a viable adaptation of the CETSA method for adherent cells. Based on the data obtained in Fig 1 and Table 2, the PBS immersion heating method was selected as the method of cell heating. Heating U2OS cells in 55°C PBS for 3 minutes followed by room temperature cooling for 5 minutes and subsequent fixation with formaldehyde (PFA) resulted in a dramatic loss (92% reduction) in cells remaining adhered to the plate bottom (Fig 2A). This was not improved by the use of poly-D-lysine coated plates or an alternative heating buffer (LCIS). In comparison, only 12% of the cells were lost when plates were fixed with either PFA followed by 100% methanol or 100% methanol alone.

Chk1 expression was determined by immunofluorescent (IF) imaging with six different anti-Chk1 antibodies (Fig 2B and Table 1). Chk1 was found to be predominantly nuclear for the majority of antibodies following PFA, PFA then MeOH or MeOH alone staining. Cellular heating to 55°C by PBS immersion reduced the Chk1 nuclear intensity following IF staining with 2G1D5, G-4, EP691Y and FL-476 antibodies with the greatest differential between heated and unheated cells observed following PFA then MeOH fixation (Fig 2C). PBS immersion heating left the cells fragile with obvious cellular damage (S2 Fig). We have, however, not exhausted all potential heating matrices / buffer and alternatives may prove more suitable.

**Fig 3:**
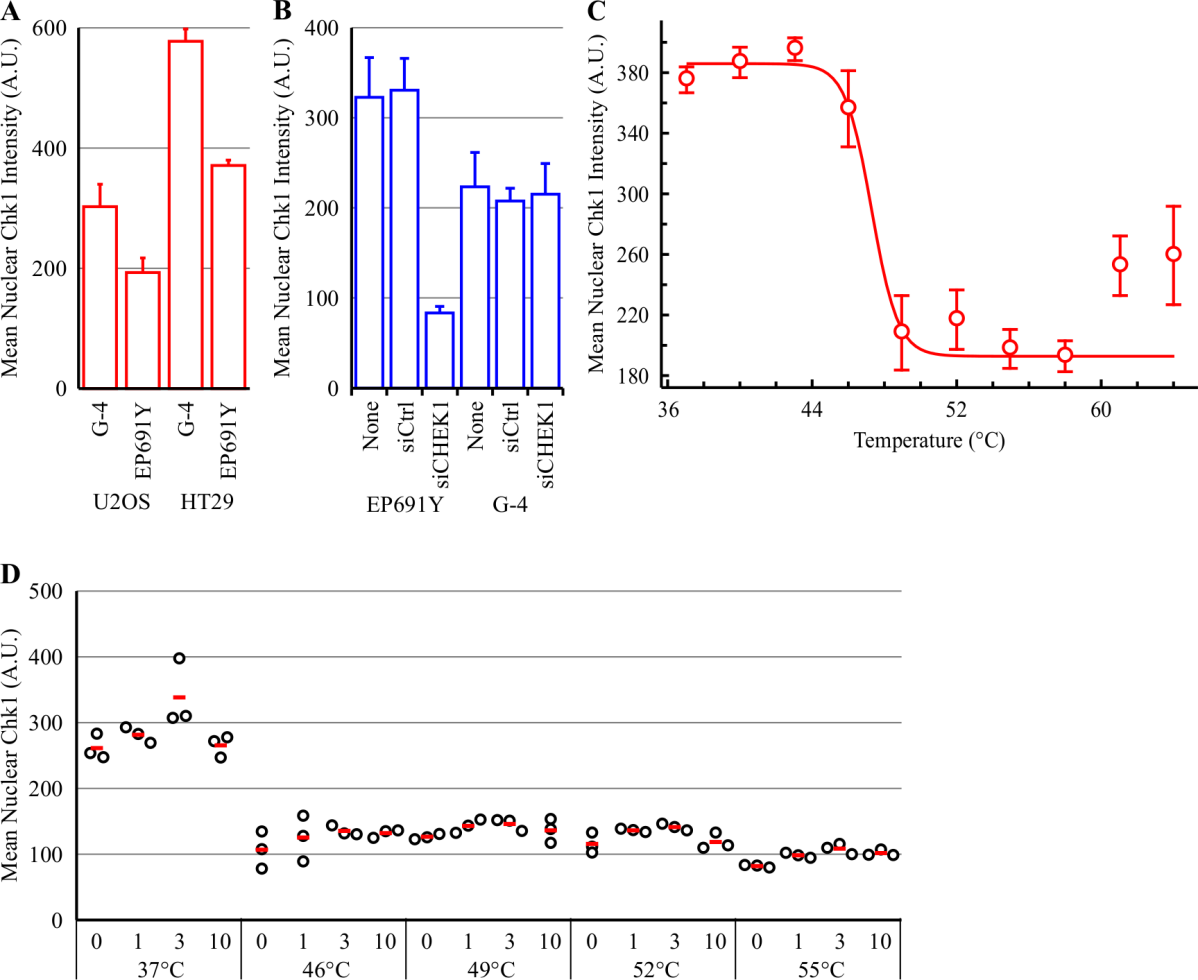
V158411 does not thermostabilize Chk1 in intact cells heated by the PBS immersion method. (A) Mean nuclear Chk1 intensity was determined in U2OS and HT29 cells following immunofluorescent staining with the anti-Chk1 antibodies G-4 and EP691Y and high content imaging. Mean of 3 independent wells ± SD. (B) HT29 cells were transiently transfected with control (siCtrl) or CHEK1 smart pool siRNA (siCHEK1) for 48 hours. Mean nuclear Chk1 intensity was determined by immunofluorescent staining with G-4 or EP691Y antibodies and high content imaging. (C) Thermal response profile of Chk1 in HT29 cells detected by immunofluorescent imaging with the EP691Y antibody following immersion in PBS pre-heated to the indicated temperature. Values are the mean of four wells ± SD. (D) HT29 cells were treated with 0–10 μM V158411 for 30 minutes before being heated by immersion in PBS pre-heated to the indicated temperature for 3 minutes followed by room temperature for 5 minutes. Nuclear Chk1 intensity was determined by immunofluorescent staining using the EP691Y antibody and high content imaging. Red line, mean.

The mean nuclear Chk1 intensity in U2OS cells was relatively weak. Comparison to HT29 cells revealed an approximately 2-fold greater Chk1 IF intensity in HT29 cells compared to U2OS cells following staining with either the G-4 or EP691Y antibodies (Fig 3A). To confirm that the G-4 and EP691Y anti-Chk1 antibodies were detecting Chk1 following PFA then MeOH staining, Chk1 was knocked down with a SMARTpool CHEK1 siRNA. Knockdown of Chk1 in HT29 cells with siRNA reduced the mean nuclear Chk1 intensity by 3.8-fold following Chk1 IF detection with EP691Y (Fig 3B). In comparison, the CHEK1 siRNA had no effect on the mean nuclear Chk1 intensity in HT29 cells stained with G-4 suggesting that this antibody was not detecting Chk1. All future IF studies were therefore conducted using the EP691Y rabbit monoclonal anti-Chk1 antibody.

The TFI_50_ (the temperature that reduced the fluorescence intensity to halfway between the baseline and maximum), was 47.1°C for Chk1 in HT29 cells heated by the PBS immersion method (Fig 3C and Table 3). This was 3.2°C lower than that determined using the thermocycler heating of HT29 cells in suspension and Chk1 detection by western blotting ([11], Table 3). Heating HT29 cells to 46, 49, 52 or 55°C by immersion in pre-heated PBS reduced the mean nuclear Chk1 intensity measured by IF (Fig 3D). Treating the cells with 1–10 μM V158411 for 30 minutes prior to PBS heating did not alter the mean nuclear Chk1 intensity.

**Table 3:** *T*_*agg*_ or TFI_50_ values for Chk1 in HT29 cells.

Heating Method	Detection Method	*T*_*agg*_ or TFI_50_ (°C)
Thermocycler	WB	50.3
Immersion in PBS	IF	47.1
Sealed in water bath,		
25 μL volume	IF	47.8
50 μL volume	IF	50.4

WB, western blot; IF, immunofluorescence

### Effects of Chk1 activation by hydroxyurea on Chk1 thermostabilization by V158411

**Fig 4:**
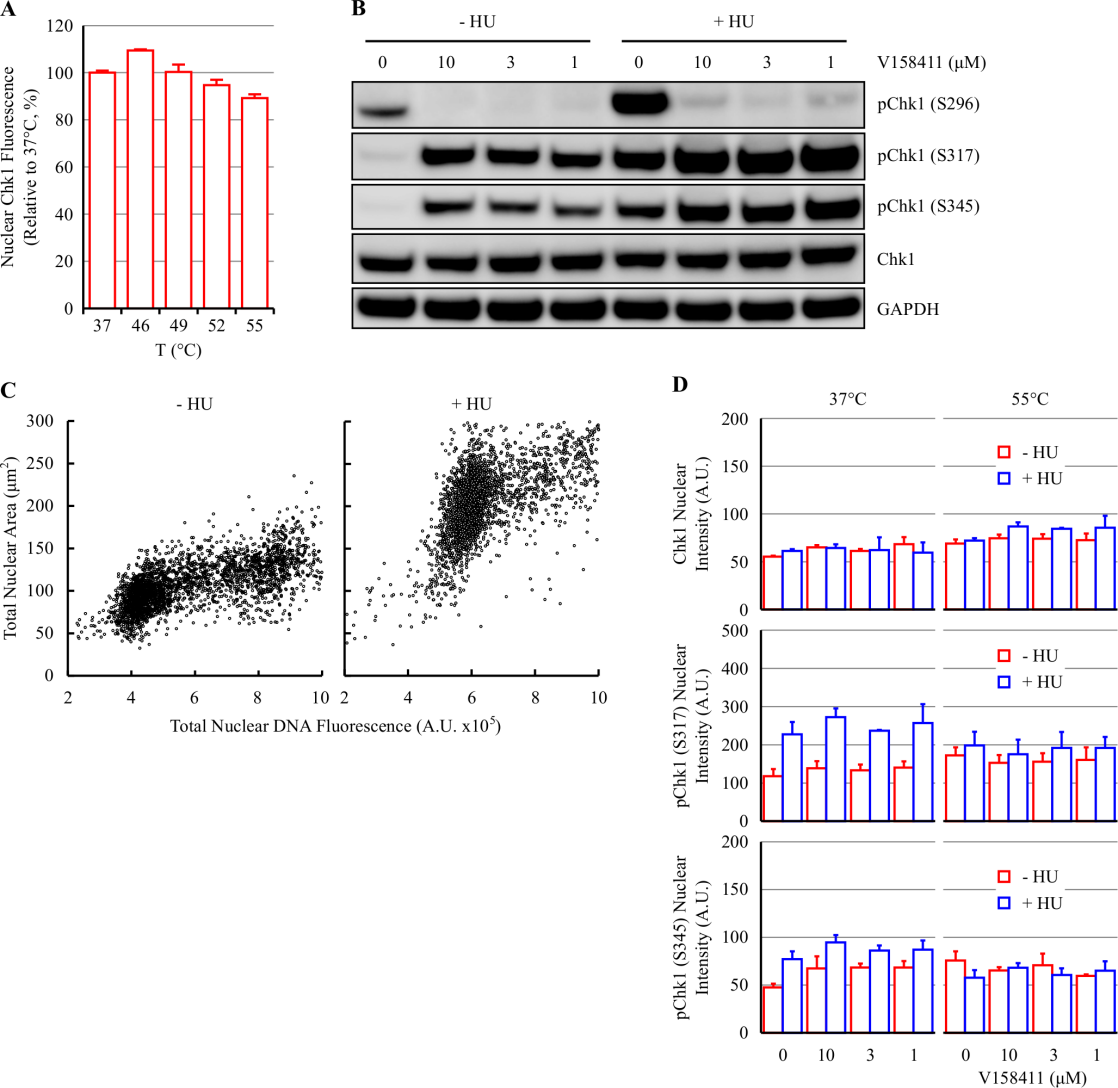
Hydroxyurea increases Chk1 activation but not thermostabilization by V158411. (A) A sealed plate containing HT29 cells was submerged and heated in a water bath pre-heated to 37–55°C. Nuclear Chk1 intensity was determined by immunofluorescent staining using the EP691Y antibody and high content imaging. Values are the mean of 3 wells ± SD. (B) HT29 cells were treated with 0 or 2.5 mM HU for 18 hours followed by 0–10 μM V158411 for 30 minutes. Protein expression levels were determined by immunoblotting. (C) HT29 cells were treated with 0 or 2.5 mM HU for 18 hours and then stained with 1 μg/ml Hoechst 33342 for 30 minutes. Cells were imaged on the Operetta and the total nuclear DNA fluorescent intensity plotted against cellular area. Red box, arrested cells. (D) HT29 cells were treated as above. Cells were heated as indicated and nuclear Chk1, pChk1 (S317) or pChk1 (S345) intensity was determined by single cell immunofluorescent imaging. Values are the mean of 3 wells ± SD.

Heating HT29 cells to temperatures between 49 and 55°C by immersion of a sealed CellCarrier 96WP in a pre-heated water bath did not reduce the mean nuclear Chk1 intensity measured by IF (Fig 4A). As the time-to-temperature was substantially longer for the sealed heating method than for PCR tubes, the plates were heated for 8 minutes rather than 3 minutes.

Chk1 kinase is activated in response to replication stress and DNA damage via the ATR dependent phosphorylation of serine 317 and 345 [12]. In the absence of S317/S345 phosphorylation, the ATP binding site of Chk1 is occluded rendering the kinase catalytically inactive [13,14]. Hydroxyurea (HU) inhibits ribonucleotide reductase thereby decreasing the production of deoxyribonucleotides leading to increased replication stress, S-phase arrest and Chk1 activation [15]. Treatment of HT29 cells with 2.5 mM HU for 18 hours increased Chk1 activation as evidenced by increased Chk1 phosphorylation on S296, S317 and S345 (Fig 4B) with 71.4 ± 0.7% (n = 6 wells ± SD) of HU treated cells exhibiting evidence of S-phase arrest (increased nuclear area, DNA content >G1, <G2) compared to 2.5 ± 1.0% of untreated cells (Fig 4C). V158411 also increased Chk1 activation but to a lesser extent than HU treatment. V158411 inhibited Chk1 autophosphorylation in HU treated and untreated cells suggestive of Chk1 target engagement under both treatment regimens.

Activation of Chk1 with HU allowed the evaluation of two additional antibodies that specifically detect active Chk1 [16]. To evaluate the effect of HU treatment on Chk1 thermostability in the absence or presence of V158411, HT29 cells were treated with 2.5 mM HU for 18 hours followed by 10 μM V158411 for 30 minutes then heated by submerging the sealed plate in a pre-heated water bath set to 55°C. A small change in nuclear levels of pS317 and pS345 detected by IF following HU and V158411 treatment prior to heating were observed. HU treatment induced a 1.9-fold and 1.6-fold increase in nuclear Chk1 pS317 and pS345 respectively (Fig 4D). Heating the cells did not alter the nuclear levels of Chk1, Chk1 pS317 or pS345 either in the absence and presence of prior HU treatment therefore V158411 treatment had no effect on the thermostabilization of total Chk1 or phosphorylated Chk1 in either HU treated or untreated cells (Fig 4D) after HU treatment and detection by IF.

### Revisiting the sealed heating method–V158411 decreases Chk1 nuclear immunofluorescence in HT29 cells heated in a low well volume

**Fig 5:**
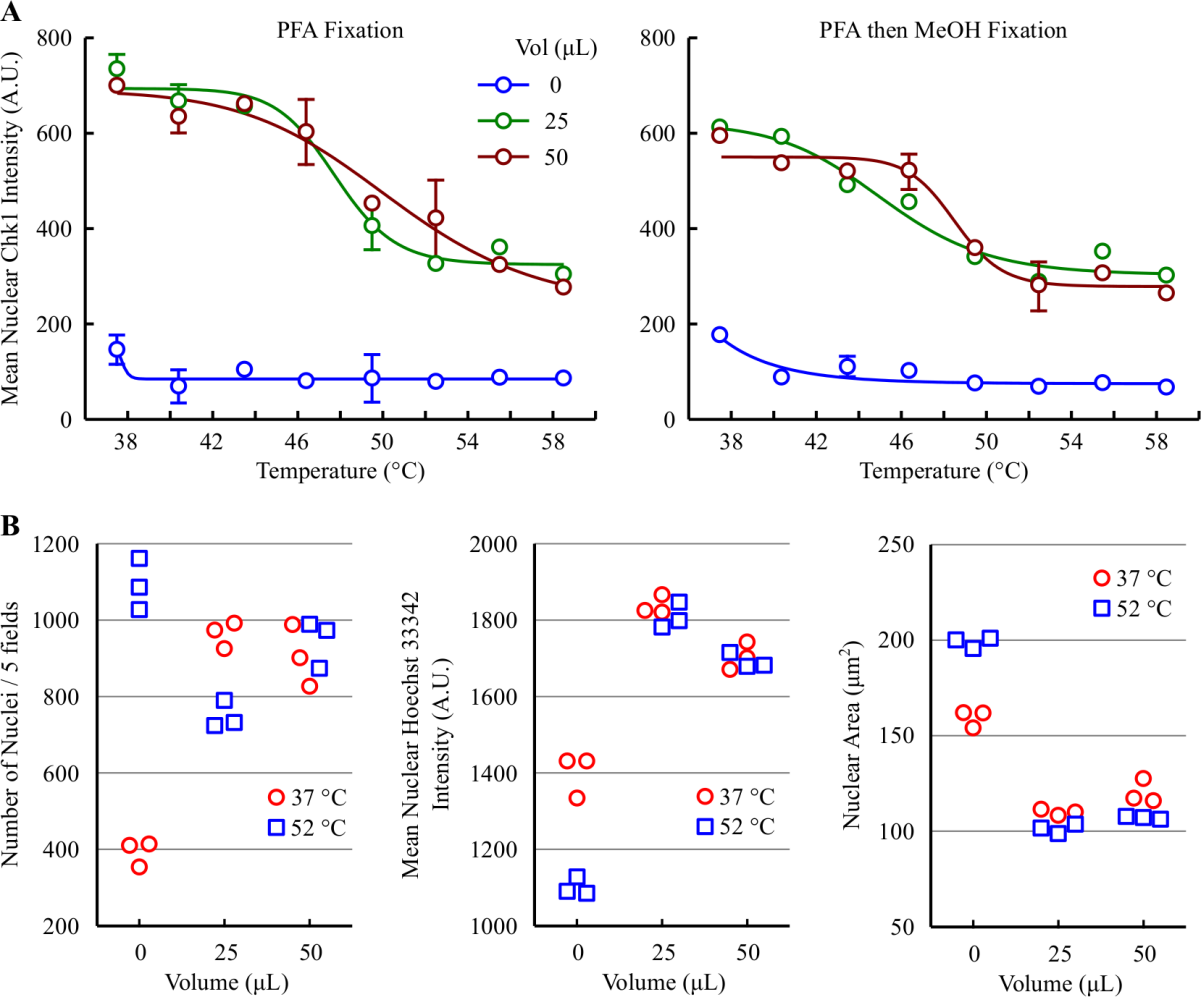
Revisiting the sealed heating method–heating cells in a low volume decreases nuclear Chk1. (A) Thermal response profile of Chk1 in HT29 cells in 0, 25 or 50 μL media detected by immunofluorescent imaging with the EP691Y antibody following heating at 37 to 58°C by floating in a pre-heated water bath and fixation with formaldehyde or formaldehyde then methanol fixation. Values are the mean of three wells ± SD. (B) Nuclei parameters were determined in Harmony software. Values represent the individual results of 3 different wells.

CellCarrier Ultra 96 well plates have a very thin bottom (190 μm thick) which should facilitate the rapid heating of cells attached to this surface. In previous experiments, heating cells in 200 μL media resulted in a long time-to-target-temperature in the well and no thermo-destabilization of Chk1. To minimize the effects of convection, we evaluated lower well media volumes (0, 25 or 50 μL) on Chk1 stability following heating. Heating the cells in a lower volume of media resulted in Chk1 destabilization with a TFI_50_ of 47.8 and 50.4°C in 25 and 50 μL volumes respectively (Fig 5A and Table 3). Heating the cells in a completely empty well was not suitable and resulted in loss of nuclear Chk1 fluorescence and cellular damage (Fig 5B and S3A Fig). Cells heated in low volumes of media were more robust than those heated by the PBS immersion method with formaldehyde fixation sufficient for cells to remain attached to the plate following IF processing. Indeed, formaldehyde fixation produced a greater signal to background window than formaldehyde followed by methanol fixation (Fig 5A).

**Fig 6:**
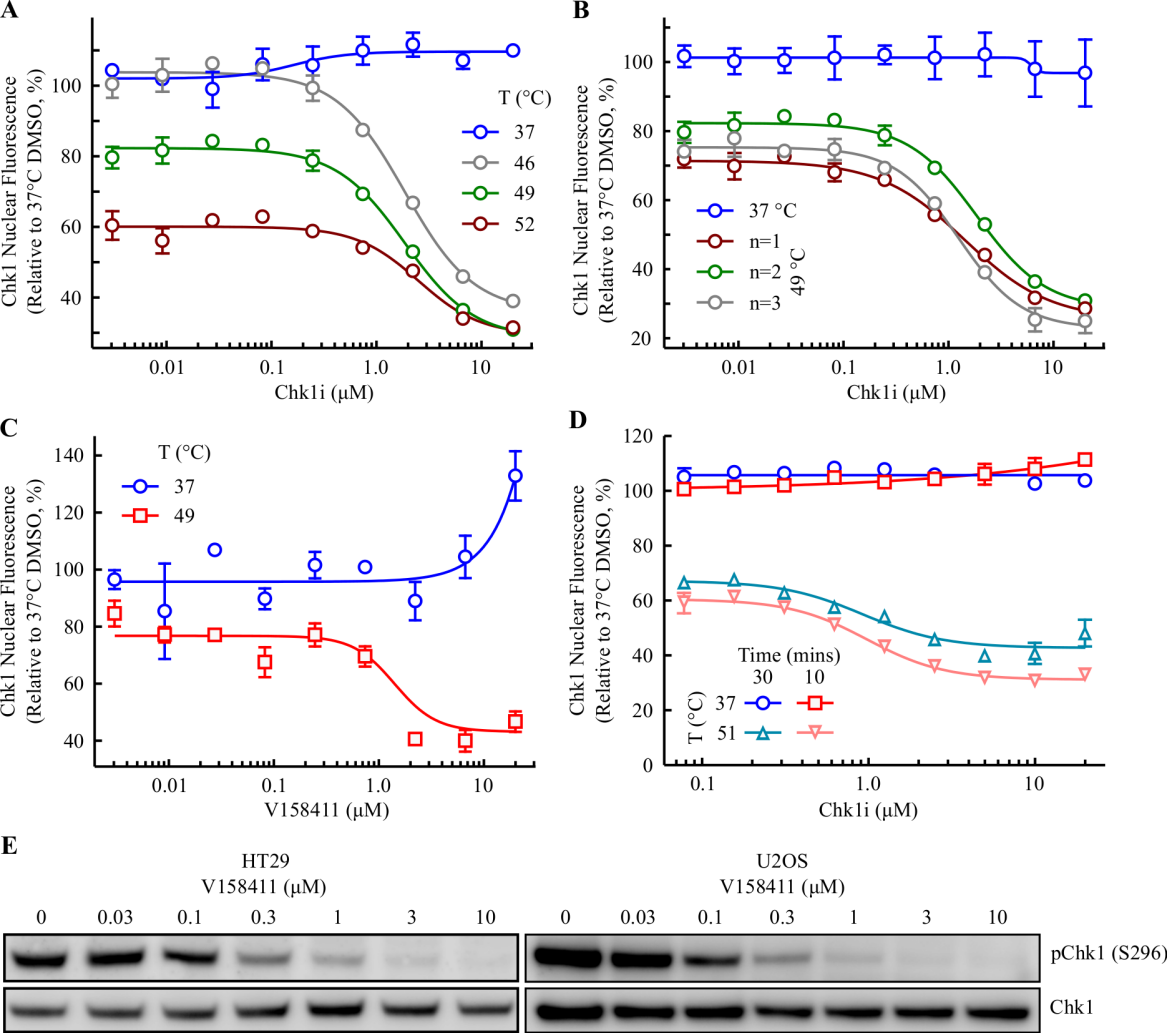
Revisiting the sealed heating method–V158411 decreases Chk1 nuclear immunofluorescence in HT29 cells heated in a low well volume. (A) HT29 cells were treated with 0–20 μM V158411 for 10 minutes in 25 μL media before being heated at the indicated temperature for 3 minutes followed by room temperature for 5 minutes. Nuclear Chk1 intensity was determined by immunofluorescent staining using the EP691Y antibody and high content imaging. Values are the mean of three wells ± SD. (B) HT29 cells were treated with 0–20 μM V158411 for 10 minutes in 25 μL media before being heated to 49°C. For 37°C, mean of n = 4 ± SD; for 49°C, dose response curves of 3 independent experiments are shown (mean of 3 wells ± SD). (C) U2OS cells were treated with 0–20 μM V158411 for 10 minutes in 25 μL media before being heated to 49°C. Mean of 3 determinations ± SD. (D) HT29 cells were treated with 0–20 μM V158411 for 10 or 30 minutes in 50 μL media before being heated to 51°C. Mean of 3 wells ± SD. (E) HT29 or U2OS cells were treated with 0–10 μM V158411 under identical conditions of cell number to media volume as (B) above for 10 minutes. Protein expression levels were determined by immunoblotting and quantified by densitometry.

To minimize the effects of evaporation we evaluated the effect of V158411 on Chk1 thermal stability following a short 10 minute incubation. Pre-incubation of HT29 cells with 0–20 μM V158411 for 10 minutes resulted in a reduction in Chk1 nuclear immunofluorescence with an EC_50_ of 1.9, 1.9 or 2.5 μM when heated to 46, 49 or 52°C respectively (Fig 6A and S3B Fig). This was in direct contrast to the CETSA classic method where V158411 increased Chk1 thermostabilization [11]. The HCIF-CETSA method was relatively robust and reproducible with EC_50_ values of 1.9, 1.9 and 1.2 μM (mean 1.7 ± 0.4 μM) determined in three independent experiments (Fig 6B). Likewise, in U2OS cells an EC_50_ value of 1.4 ± 0.3 μM was determined (Fig 6C) though the signal to background was much lower in this cell line. In the unheated U2OS cells, V158411 treatment increased the mean nuclear Chk1 fluorescence coupled with a concomitant decrease in the mean cytoplasmic Chk1 fluorescence (S4 Fig). Chk1 can shuttle between the nucleus and cytoplasm [17] including in response to DNA damage. Single agent Chk1 inhibition induces DNA damage and the effect observed may be the shuttling of Chk1 to the nucleus in response to this damage. In addition, the relatively low Chk1 immunofluorescence signal in the U2OS cell line is most likely magnifying the effect compared to the HT29 cell line. The EC_50_ values generated by HCIF-CETSA were in good concordance with the CETSA classic EC_50_ values of 3.3 ± 3.4 μM and 2.3 ± 1.2 μM in HT29 and U2OS cells respectively.

**Fig 7:**
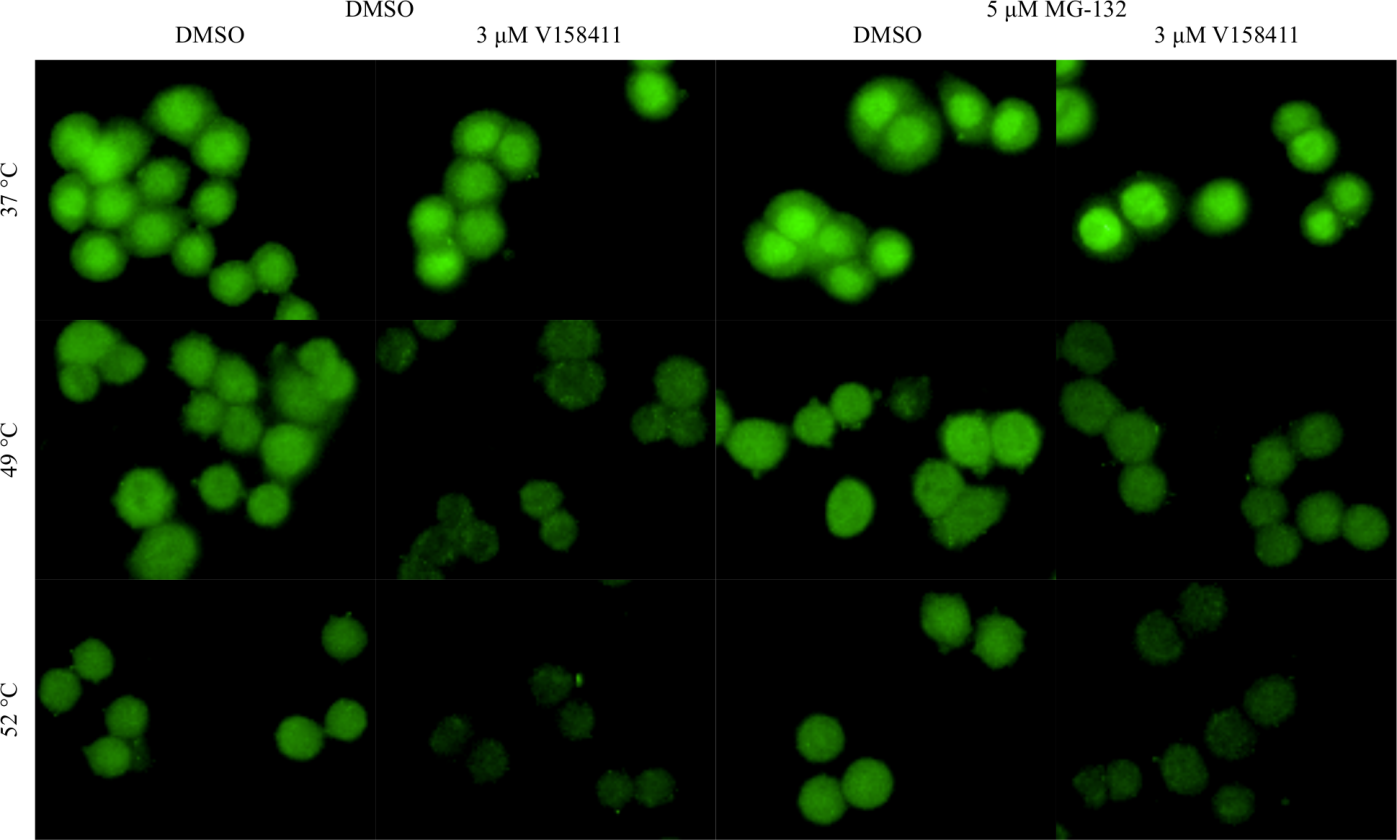
Inhibition of the proteasome does not block Chk1 thermal destabilization. HT29 cells were treated with 5 μM MG-132 for 2 hours then with 0 or 3 μM V158411 for 10 minutes in 25 μL media before being heated to 49 or 52°C. Cells were imaged using the Operetta with a 40x high NA objective.

Incubating the cells with V158411 for a longer time (30 minutes) in a larger volume (50 μL, to minimize evaporation effects) had little effect on the HCIF-CETSA EC_50_ value with EC_50_ values of 1.0 and 1.2 μM obtained following a 10 and 30 minute incubation respectively (Fig 6D). Under identical cellular conditions to the HCIF-CETSA, V158411 reduced Chk1 autophosphorylation (pS296) in HT29 or U2OS cells with an IC_50_ value of approximately 0.16 and 0.11 μM respectively (Fig 6E). Inhibiting the proteasome with MG-132 did not block the temperature induced decrease in Chk1 nuclear fluorescence in HT29 cells pre-treated with either DMSO or V158411 nor did it alter the sub-cellular localization in either unheated or heated cells (Fig 7).

### Structurally distinct Chk1 inhibitors decrease Chk1 nuclear fluorescence in HT29 cells following low well volume heating

**Fig 8:**
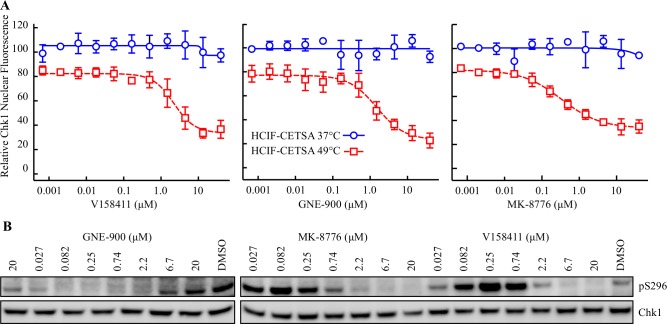
Structurally distinct inhibitors of Chk1 decrease Chk1 nuclear fluorescence following heating in a low well volume. (A) HT29 cells were treated with 0–40 μM Chk1 inhibitor for 10 minutes in 25 μL media before being heated at the indicated temperature for 3 minutes followed by room temperature for 5 minutes. Nuclear Chk1 intensity was determined by immunofluorescent staining using the EP691Y antibody and high content imaging. (B) HT29 cells were treated with 0–20 μM Chk1 inhibitor using the same dilution series and under identical conditions of cell number to media volume as (A) above for 10 minutes. Protein expression levels were determined by immunoblotting and quantified by densitometry.

We further determined the propensity of two additional structurally diverse Chk1 inhibitors (GNE-900 [18]and MK-8776 [19], S5 Fig) to increase Chk1 thermal destabilization and inhibit Chk1 autophosphorylation. GNE-900, MK-8776 and V158411 all induced a large decrease in nuclear Chk1 fluorescence following heating to 49°C with EC_50_ values of 1.3 ± 0.3, 0.32 ± 0.1 and 2.0 ± 0.9 μM respectively (Fig 8A). The same compound dilutions were used to determine the effect of the Chk1 inhibitors on Chk1 autophosphorylation under the HCIF-CETSA treatment conditions. All three compounds reduced S296 phosphorylation (Fig 8B) with IC_50_s of 0.50, 0.44 and 1.2 μM for GNE-900, MK-8776 and V158411 suggestive of Chk1-inhibitor target engagement within the 10 minute incubation period.

## Discussion

The measurement of drug-protein interactions in living cells and ultimately the target tissue is fundamental to the drug discovery process. The usage of CETSA [1] to measure drug-target interaction has increased dramatically since its first description in 2013. CETSA has the potential to work on most targets in a wide variety of cell types as it requires no protein modification or the generation of tracer molecules, assays the target protein at the endogenous level, requires simple equipment to perform and when western blotting is used for detection, has a wide range of detection antibodies available. However, the assay format described in these papers has several potential drawbacks namely cells have to be heated in suspension, in PBS, at relatively high densities. For adherent cells, heating cells in suspension is an obvious drawback and the process of trypsinization and resuspension may alter cellular physiology and target pharmacology. Additionally, having to treat cells at high cell densities may result in an underestimation of target engagement potency and make comparisons to downstream pharmacology assays more difficult. We have therefore developed a novel cellular target engagement assay in adherent live cells using the principle of ligand-induced changes to protein thermal stability coupled with high-content single cell immunofluorescent imaging in an attempt to mitigate some of these potential liabilities.

Development of this high content platform based target engagement assay was more technically challenging than anticipated. Problems that required overcoming included (i) heating a 96WP, (ii) accurately measuring in well temperature changes, (iii) detection of target protein and (iv) validation of assay methodology. Chk1 kinase and the potent, selective small molecule inhibitor V158411 [9,10] was selected as an assay test system. We have previously demonstrated that using the assay conditions described in Jafari *et al*, [2] V158411 increased the thermal stabilization of Chk1 in HT29 and U2OS cells [11]. Whilst heating cells by the PBS immersion method appeared to most closely replicate the heating conditions achieved in a PCR tube and resulted in a temperature dependent decrease in nuclear Chk1 immunofluorescence there was no effect of V158411 on Chk1 nuclear immunofluorescence. Heating cells by the PBS immersion method resulted in significant cellular damage (S2 Fig) which may, in part, account for the lack of additional thermal stabilization / destabilization observed in the presence of V158411. Conversely, covering the cells with 200 μL PBS, sealing the plate and then heating by immersion in a pre-heated water bath did not alter Chk1 nuclear fluorescence in the presence or absence of V158411. This was despite the fact that CellCarrier plates have a very thin (0.180 μm) bottom and most likely reflects that heating could only be achieved through the bottom (and top for the submerged plate) and not the well sides with in-well convection therefore reducing the rate of temperature rise. Heating cells by floating in a water bath where the well volume had been reduced to 25 μL resulted in a temperature dependent decrease in nuclear Chk1 fluorescence that was further reduced by the pre-incubation of cells with V158411. The EC_50_ values for V158411 determined by this method were approximately equivalent to the EC_50_ values determined using the CETSA classic method suggesting a reasonable correlation between the two methods. The HCIF-CETSA method appeared robust with good inter-assay reproducibility. The EC_50_ values determined by CETSA classic or HCIF-CETSA were 2.7–23-fold greater than the autophosphorylation IC_50_. The CETSA classic or HCIF-CETSA EC_50_ values appeared to tally more closely with changes in Chk1 inhibitor dependent cellular pharmacology and may reflect the cellular biology of Chk1 kinase.

The reasons behind the difference in Chk1 protein stability following binding by V158411 between the CETSA classic (stabilization) and the HCIF-CETSA method (destabilization) were not immediately apparent. However, it should be noted that the assays have very different conditions / formats and have different but related endpoints. CETSA classic determines the amount of soluble protein remaining following heating whilst HCIF-CETSA determines the loss of an antibody epitope. The interpretation of the immunofluorescence results may be complicated by the choice of antibody used. Cell fixation can result in protein denaturation and therefore the antibodies used in immunofluorescence may not recognize a native state protein but an epitope that is revealed following partial denaturation of the protein by the fixation process. The subsequent heat denaturation and unfolding induced during the HCIF-CETSA process may increase the recognition of the target protein by the antibody utilized in the assay. Therefore selecting an antibody that faithfully reports the protein state may be critical for the HCIF-CETSA method. Utilizing alternative protein detection methods (such as those discussed later) may overcome some of these limitations.

This assay format has some distinct advantages over the CETSA classic methodology first described by Molina *et al*. [1] namely adherent cells can be heated whilst still attached at relatively low cell densities. As the method uses standard format 96 well plates (and potential miniaturization to 384 well plates), it is readily amenable to scale, automation and laboratory workflows. If longer compound incubations are necessary for target engagement to occur, the cells can simply be incubated in a larger well volume for the required time then the media volume reduced and the cells heated. Adaptation of the CETSA classic method to homogenous plate based methodologies (such as AlphaScreen or ELISA [3], referred to as HT-CETSA) has the potential to increase the throughput of this assay format. In the HCIF-CETSA method, it is more difficult to control the cell heating than the thermocycler method. However, by using the same water bath coupled with precise working, it is possible to produce highly reproducible data.

Another drawback of using immunofluorescence for target detection is that the number of good quality, well validated antibodies available is much lower than that for western blotting. The CETSA classic method has been demonstrated to work with reporter tagged proteins for example (i) using Enzyme Fragment Complementation (EFC) between two inactive b-galactosidase fragments (InCELL Hunter Target Engagement Assay, DiscoveRx) or (ii) using a Nanoluc-tagged protein (Promega). This approach has recently been utilized to develop a high-throughput dose-response cellular thermal shift assay (HTDR-CETSA) based on BacMam titratable expression of ePL tagged full-length target proteins [20]. The possibility therefore exists to generate and detect proteins tagged with a fluorescent protein (e.g. EGFP), a fluorescent reporter (e.g. HaloTag or SNAP-tag) or a protein tag for which high quality IF compatible antibodies are available (e.g. HA or FLAG tag). CRISPR technology now allows in principle gene specific protein tagging at either the N- or C-terminus [21,22]. This technology could be used to generate a cell line containing a reporter-protein fusion protein expressed at endogenous levels to measure target engagement.

In summary, the method described here allows the high throughput determination of target engagement in adherent cells based on the principle of altered protein thermal stabilization / destabilization in response to ligand binding and is therefore a valuable advance in the CETSA method. It should serve as a valuable tool for those engaged in small molecule drug development.

## Supporting information

S1 Fig Example of Papago thermometer set up. Papago thermometer WIFI module with two k-type thermocouples placed in CellCarrier plate and heated by immersion in Grant W14 water bath.
(TIF)

S2 Fig Heating of cells by the PBS immersion method induces cell damage. HT29 or U2OS cells were heated by immersion in PBS pre-heated to 37 or 55°C and fixed with formaldehyde them methanol.
(TIF)

S3 Fig Effects of cellular heating on attached HT29 cells in low volume media. (**A**) Example images of HT29 cells growing attached on a CellCarrier Ultra 96WP in 0, 25 or 50 μL media heated to 37 or 52°C. Chk1 was detected by immunofluorescence with the anti-Chk1 antibody EP691Y and imaged with a 20x objective on an Operetta HC imager. (**B**) Example images of HT29 cells treated with 0–20 μM V158411 for 10 minutes then heated to 37 or 49°C.
(TIF)

S4 Fig V158411 induces Chk1 cytoplasm to nuclear translocation in U2OS cells. U2OS cells were treated with 0–20 μM V158411 for 10 minutes in 25 μL media. Data is from Fig 6C. Mean nuclear and cytoplasmic Chk1 fluorescence intensity was determined using Harmony software.
(TIF)

S5 Fig Chemical structures of Chk1 inhibitors. (TIF)
