# Supplementary figures and images for: A high content, high throughput cellular thermal stability assay for measuring drug-target engagement in living cells
Source: PLoS One. 2018 Apr 4;13(4):e0195050. doi: 10.1371/journal.pone.0195050 (PMC5884524; doi:10.1371/journal.pone.0195050)

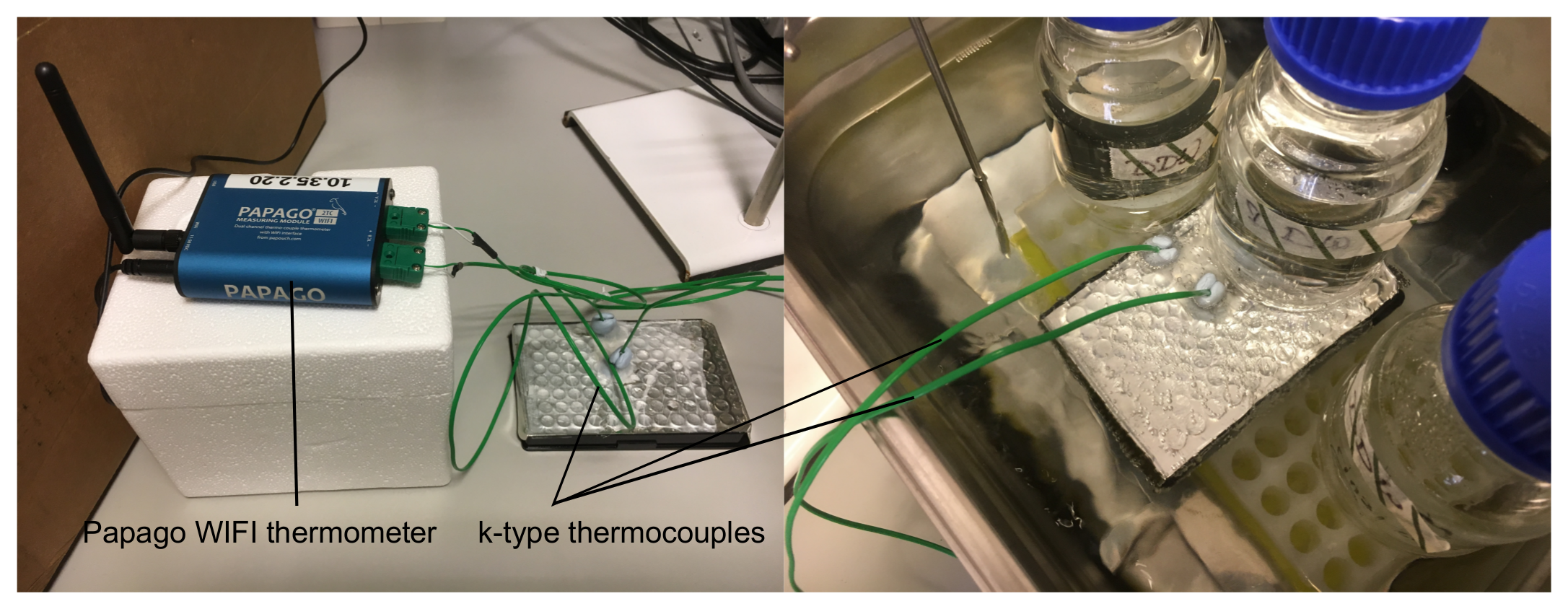

Supplement: S1 Fig — Papago thermometer WIFI module with two k-type thermocouples placed in CellCarrier plate and heated by immersion in Grant W14 water bath. (TIF) [file pone.0195050.s001.tif]

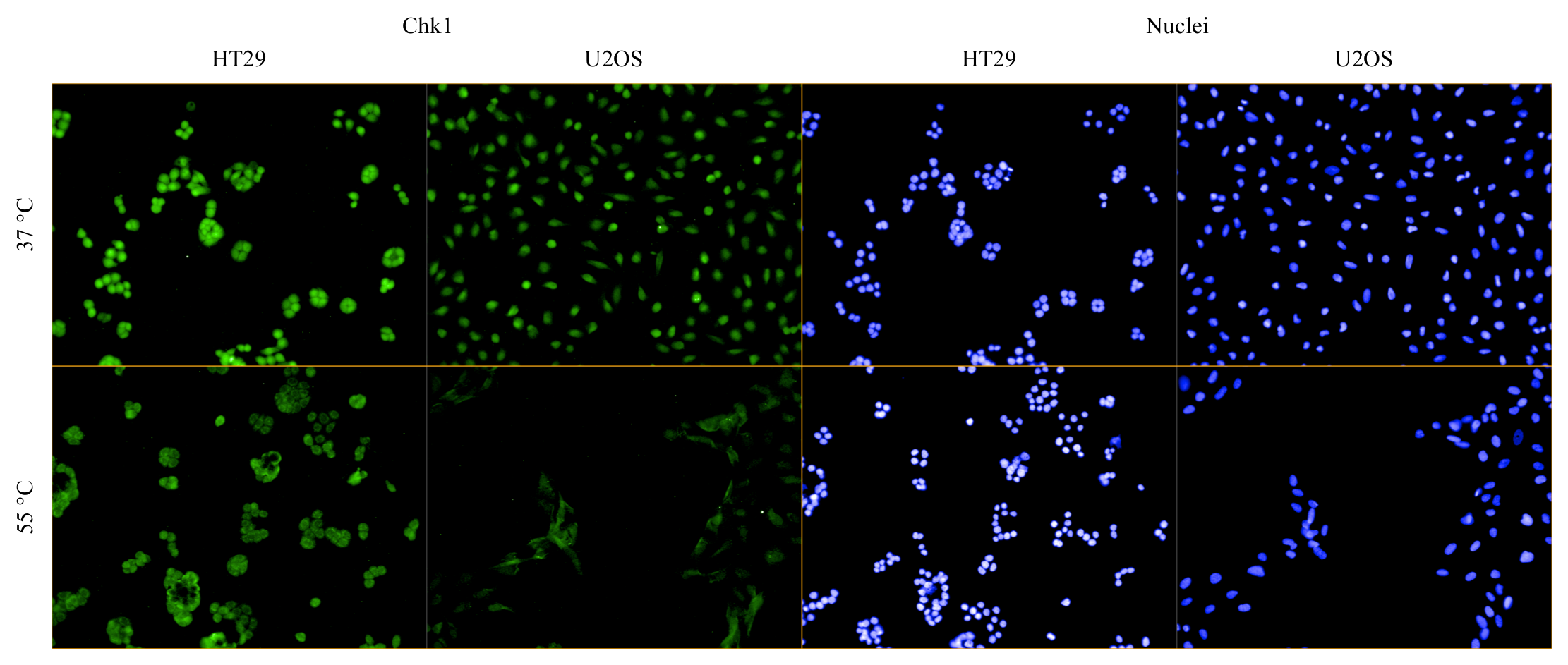

Supplement: S2 Fig — HT29 or U2OS cells were heated by immersion in PBS pre-heated to 37 or 55°C and fixed with formaldehyde them methanol. (TIF) [file pone.0195050.s002.tif]

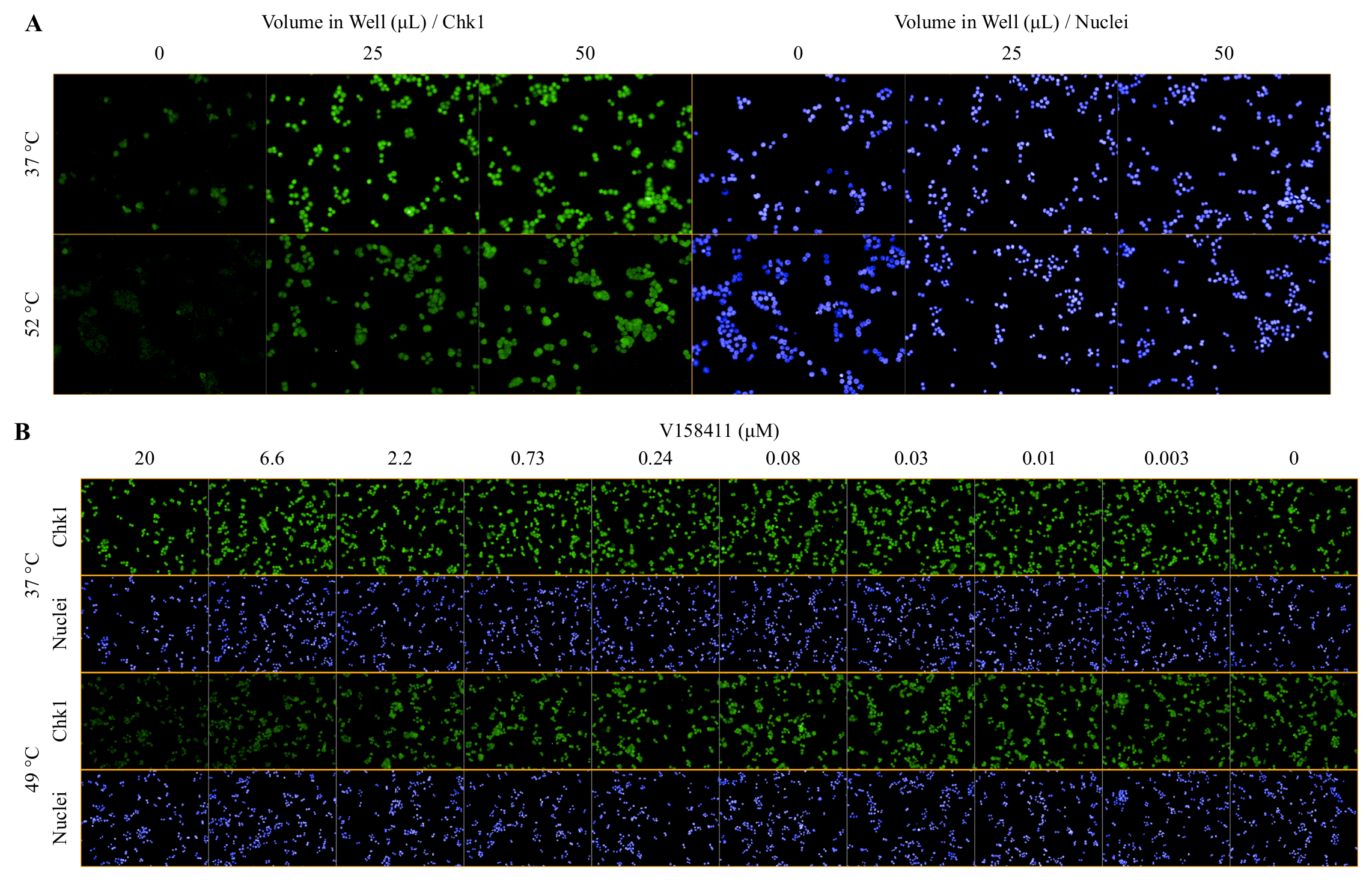

Supplement: S3 Fig — (A) Example images of HT29 cells growing attached on a CellCarrier Ultra 96WP in 0, 25 or 50 μL media heated to 37 or 52°C. Chk1 was detected by immunofluorescence with the anti-Chk1 antibody EP691Y and imaged with a 20x objective on an Operetta HC imager. (B) Example images of HT29 cells treated with 0–20 μM V158411 for 10 minutes then heated to 37 or 49°C. (TIF) [file pone.0195050.s003.tif]

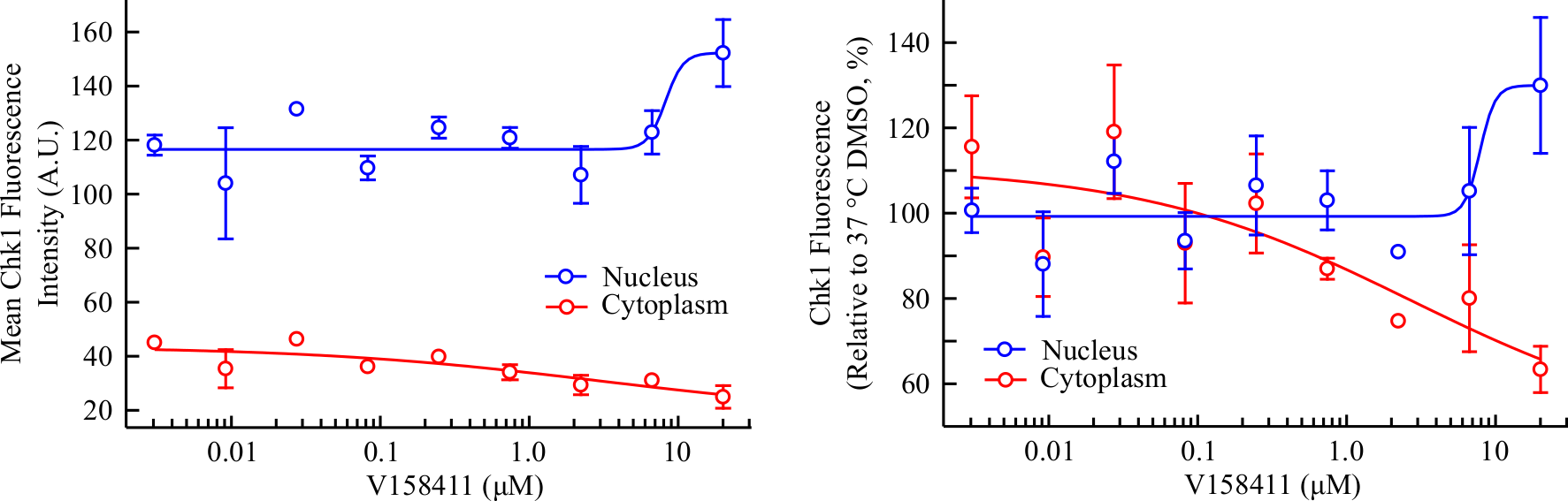

Supplement: S4 Fig — U2OS cells were treated with 0–20 μM V158411 for 10 minutes in 25 μL media. Data is from Fig 6C. Mean nuclear and cytoplasmic Chk1 fluorescence intensity was determined using Harmony software. (TIF) [file pone.0195050.s004.tif]

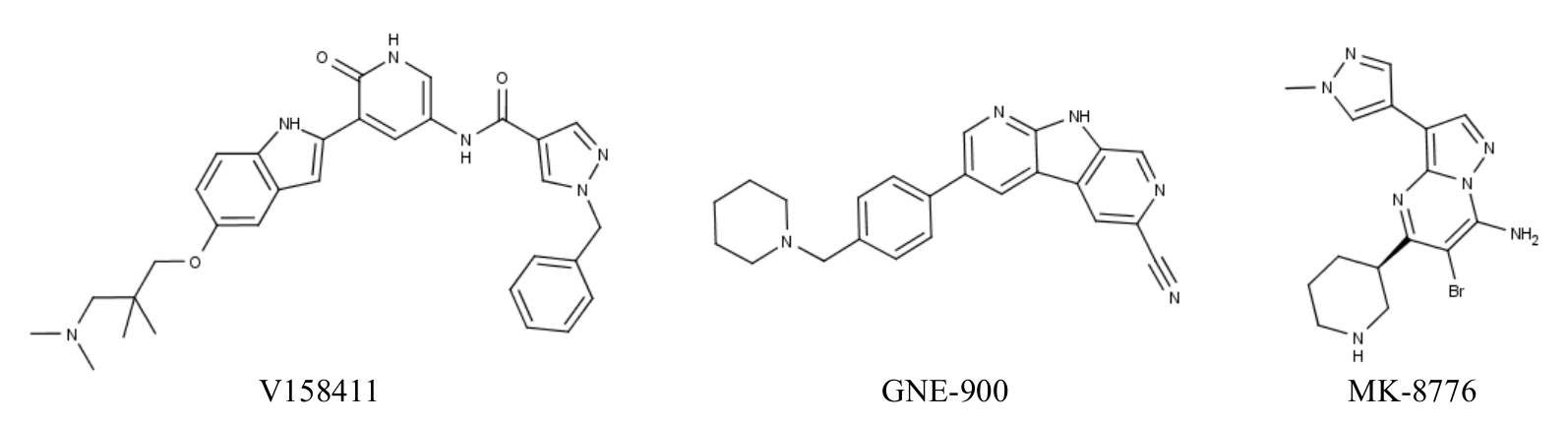

Supplement: S5 Fig — (TIF) [file pone.0195050.s005.tif]
